# Fire Propagation Performance of Intumescent Fire Protective Coatings Using Eggshells as a Novel Biofiller

**DOI:** 10.1155/2014/805094

**Published:** 2014-07-17

**Authors:** M. C. Yew, N. H. Ramli Sulong, M. K. Yew, M. A. Amalina, M. R. Johan

**Affiliations:** ^1^Department of Civil Engineering, Faculty of Engineering, University of Malaya, 50603 Lembah Pantai, Kuala Lumpur, Malaysia; ^2^Centre of Advanced Materials, Department of Mechanical Engineering, Faculty of Engineering, University of Malaya, 50603 Lembah Pantai, Kuala Lumpur, Malaysia

## Abstract

This paper aims to synthesize and characterize an effective intumescent fire protective coating that incorporates eggshell powder as a novel biofiller. The performances of thermal stability, char formation, fire propagation, water resistance, and adhesion strength of coatings have been evaluated. A few intumescent flame-retardant coatings based on these three ecofriendly fire retardant additives ammonium polyphosphate phase II, pentaerythritol and melamine mixed together with flame-retardant fillers, and acrylic binder have been prepared and designed for steel. The fire performance of the coatings has conducted employing BS 476: Part 6-Fire propagation test. The foam structures of the intumescent coatings have been observed using field emission scanning electron microscopy. On exposure, the coated specimens' B, C, and D had been certified to be Class 0 due to the fact that their fire propagation indexes were less than 12. Incorporation of ecofriendly eggshell, biofiller into formulation D led to excellent performance in fire stopping (index value, (*I*) = 4.3) and antioxidation of intumescent coating. The coating is also found to be quite effective in water repellency, uniform foam structure, and adhesion strength.

## 1. Introduction

Intumescent fire protective coatings have been widely used as passive fire protection (PFP) in parts of cars, airplanes, steel structures, and building components to ensure the fire safety and building regulations in many countries. Intumescent coating is fire retardant and fire resistant material, which has gained wide acceptance in the world for fire protection. This coating is used to achieve PFP for such applications as fire stopping and fireproofing to reduce the devastating cost of fire in terms of both life hazard and property damage.

Structural steel loses its load carrying ability to about 60 percent when its temperature exceeds 500°C in a fire, and this is mainly attributed to high thermal conductivity, low specific heat, and faster degradation of strength and elastic modulus of steel. When heated to about 800°C, the steels solidity decreases further to about 10 percent of its normal value [1, 2]. Intumescent coatings are designed to perform under severe conditions and to maintain the steel integrity in the event of a fire [3–5]. Many researchers have extensively studied the properties of intumescent coatings in terms of fire protection, adhesion strength, thermal stability, and water resistance [6–14]. The formulation of the coating has to be optimized in terms of physical and chemical properties in order to retard the heat transfer and ignition on the steel.

Intumescent coatings were composed of three halogen-free flame-retardant additives: an acid source (such as phase II ammonium polyphosphate, APP), a carbon source (such as pentaerythritol, PER), and a blowing agent (such as melamine, MEL) mixed together with flame-retardant fillers and polymer binder. Intumescent coating expands when exposed to a sufficiently high temperature (e.g., in a fire). The heated coating forms a porous char and produces residues that are swelled by escaping noncombustible gases to establish a protective barrier against oxygen. A combustion residue can be efficiently puffed up in order to produce tough foam over the surfaces to protect the substrate [15]. Significantly using flame-retardant fillers, such as aluminium hydroxide (Al(OH)_3_) and magnesium hydroxide (Mg(OH)_2_) replacement of conventional flame-retardants, is a realistic and promising way to overcome fire propagation and surface spread of flame because of their low flame retarding efficiency  [16–19]. The performance of the intumescent system depends on the choice of the ingredients and their appropriate combinations.

These research studies have pointed out a useful biofiller derived from chicken eggshell (CES) waste and its potential role in the fire protective coating industry. CES waste is an industrial byproduct, and its disposal constitutes a serious environmental hazard. It is known that CES waste contains about 95% calcium carbonate in the form of calcite and 5% organic materials such as type X collagen, sulfated polysaccharides, and other proteins [20, 21]. CES can be utilized in commercial products to create new value in these waste materials, and it has been highlighted recently because of its reclamation potential. Although there have been several attempts to use CES components for various applications  [22–26], its chemical composition and availability makes CES a potential source of biofiller reinforced biopolymer composites giving additional or improved thermal and mechanical properties to the coating [27]. The other advantages of using CES are that it is available in bulk quantity, lightweight, high thermal stability, and being inexpensive and environmentally friendly.

This paper is concerned with the development and evaluation of intumescent coatings properties. The efficiency of intumescent coatings on steel is investigated following the BS 476: Part 6-Fire propagation test. Moreover, the water resistance, thermal stability, char formation, and adhesion strength of intumescent coatings were also studied and evaluated.

## 2. Experimental

### 2.1. Materials and Method

Chicken eggshells (CES) were used as a biofiller in this study. The CES membrane was removed and discarded. The CES were then cleaned thoroughly and dried at 90°C for 12 h in the oven. The dried eggshells were mechanically triturated to a powder form and then milled at a milling speed of 280 rpm for 48 h, respectively, in a four-roll mill to obtain mean particle sizes of 22.99 *μ*m. The acrylic resin composed of acrylic acid, methacrylic acid, esters of these acids, or acrylonitrile was supplied by Shinko Paint & Chemical Sdn. Bhd. Ammonium polyphosphate phase II (*n* > 1000), melamine, and pentaerythritol were purchased from International Chemical Ltd, China. Titanium dioxide (R706), aluminium hydroxide, and magnesium hydroxide were purchased from Scientific Group Sdn. Bhd., Malaysia.

Intumescent fire protective coatings were prepared using acrylic binder, pigment, and flame-retardant ingredients. The formulations were prepared using high-speed disperse mixer and the viscosity of the coatings was adjusted to 95–105 KU. The compositions of intumescent coatings are given in Table 1. The prepared coating was coated on Q235 steel plate using a gun sprayer. For obtaining effective fire retardancy, the thickness of the intumescent coatings has been measured and maintained.

### 2.2. Fire Propagation Test

The fire propagation test was performed in accordance to the procedure specified in BS 476: Part 6:1989 + A1: Fire propagation [28]. The test method consists of exposing the product to a row of small flames for 20 minutes and an additional impressed irradiance of 2 kW from third to the final minute of the test. The temperature of the evolved combustion products is recorded. Furthermore, compared to the temperatures generated from a noncombustible specimen, the result is expressed as fire propagation index that provides a comparative measure of the contribution to the growth of fire made by essentially flat material, composite, or assembly. The coated face of the specimens was exposed to the heating conditions of the test. To be Class 0 certified the fire propagation index (*I*) must be ≤12. The lower the numerical value of the index is the better the material it indicates. The details of the test procedures on index of performance of specimens and calculation of results are explained as follows.

In this test, the rate and amount of heat evolved by the specimen were taken into account while it was heated in an enclosed space under prescribed conditions. Specimens of steel plate (225 × 225 × 2.3 mm) were coated with intumescent coatings (thickness of 1.5 ± 0.2 mm) and the index of performance was determined from the following equations:

(1) $$\begin{array}{c} i_{1}=\overset{t=3}{\underset{t=0.5}{\sum }}\frac{\theta_{m}-\theta_{c}}{10t}\\ i_{2}=\overset{t=10}{\underset{t=4}{\sum }}\frac{\theta_{m}-\theta_{c}}{10t}\\ i_{3}=\overset{t=20}{\underset{t=12}{\sum }}\frac{\theta_{m}-\theta_{c}}{10t}\\ I=i_{1}+i_{2}+i_{3}, \end{array}$$

where *I* is the index of performance, *t* is the time (min) from the origin at which readings were taken, *θ*_*m*_ is the temperature (°C) of the material at time *t*, and *θ*_*c*_ is the temperature (°C) of the calibration curve at time *t*.

### 2.3. Thermogravimetric Analysis

Thermogravimetric analyser (TGA) was carried out at 20°C/min under air flow in the temperature range of 30–1000°C using a TGA/SDTA851e model to study the thermal degradation and determine the residual weight of the coatings.

### 2.4. Field Emission Scanning Electron Microscopy

Microscopic analyses were carried out using a field emission scanning electron microscope (FESEM) GEMINI FESEM to examine the surface morphology of the intumescent char layers. For FESEM observation, low beam energy of 1 kV was operated to reduce the possibility of any thermal damage to the char layers.

### 2.5. Static Immersion Test

Static immersion test is considered as a standard method that evaluates water resistance of thin films using the gravimetric method. Samples of films (dimensions: 20 × 10 × 0.5 ± 0.1 mm) were immersed in distilled water at 25°C. At specific time intervals, the samples were removed and were blotted with a piece of paper towel to absorb excess water on the surfaces. Weight change was calculated by (2) and expressed as a function of time:

(2) $$\begin{array}{c} E_{\text{sw}}=\frac{\left(W_{e}-W_{o}\right)}{W_{o}}\times 100\%, \end{array}$$

where *E*_sw_ is the water uptake ratio of the film, *W*_*e*_ denotes the weight of the film at different times, and *W*_*o*_ is the dry weight of the sample.

### 2.6. Adhesion Strength

**Figure 1:**
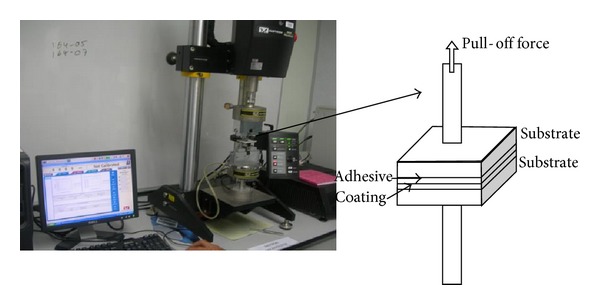
The setup for the measurement of adhesion strength.

The adhesion strength of the coated sample was determined by using the Instron Micro Tester equipment. A scheme of the adhesion strength measurement setup is shown in Figure 1.

The coatings were each sprayed on one side of 50 × 50 × 2.6 mm steel plates with a film thickness of 0.5 ± 0.05 mm. The steel plate with a dry film was attached to a bare steel plate (dimensions: 50 × 50 × 2.6 mm) using epoxy glue (thickness of 0.5 ± 0.05 mm). Then the two steel plates were then continually drawn apart in tensile mode at a constant rate of 1 mm/min using the testing device until the coating on the steel plate cracked. Adhesion strength (*f*_*b*_) in MPa was calculated based on the following equation:

(3) $$\begin{array}{c} f_{b}=\frac{F}{A}, \end{array}$$

where *f*_*b*_ is the adhesion strength, MPa; *F* is the crack charge, N; *A* is the sticking area, mm^2^.

## 3. Results and Discussion

### 3.1. Fire Propagation

In the previous study, all the samples had satisfied Class 1 prescribed in the BS 476: Part 7 surface spread of flame test. The results of subindex and index of performance of fire propagation test are as illustrated in Table 2.

The fire propagation results showed that the subindexes (*i*_1_ : *i*_2_ : *i*_3_) of samples A, B, C, and D were (1.8 : 15 : 5.7), (0 : 8.4 : 3.4), (0.2 : 8.3 : 2.9), and (0 : 3.8 : 0.7), respectively. However, the indexes of performance of samples A, B, C, and D were 22.3, 11.5, 11.2, and 4.3, respectively. In this test, only sample A is not certified to be Class 0 due to the fact that its fire propagation index was 22.3 (*I* > 12).

**Figure 2:**
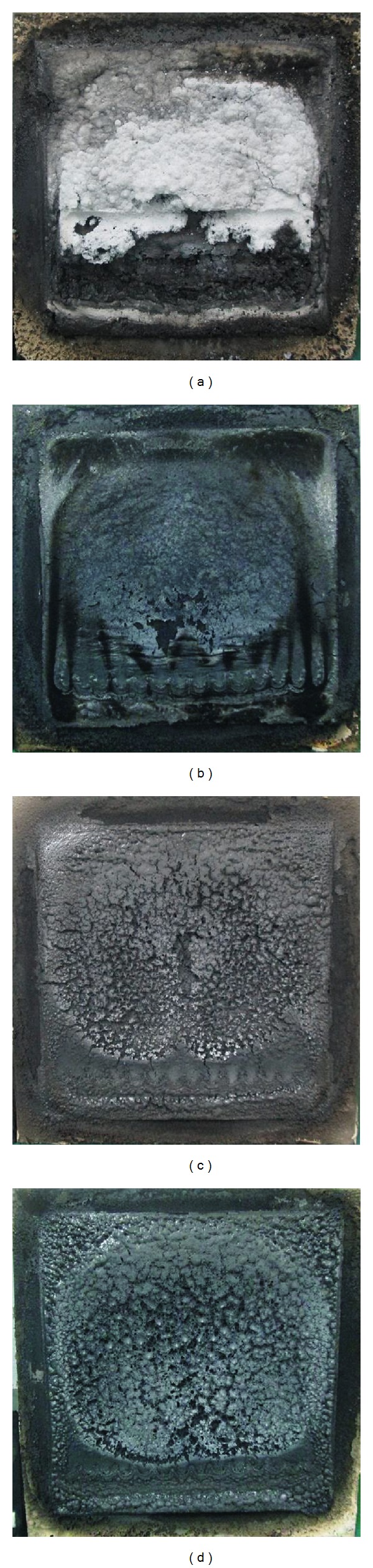
Tested samples (a) A, (b) B, (c) C, and (d) D according to BS 476: Part 6-Fire propagation test.

The tested samples according to BS 476 Part 6: Fire propagation test are as shown in Figure 2. By comparing the results of fire propagation index of samples A, B, and C, it was found that the sample C ((*I*) = 11.2) with the addition of CES has a much lower fire propagation index than that of samples A ((*I*) = 22.3) and B ((*I*) = 11.5). Incorporation of CES into the formulation (sample C) was significantly inhibited by the fire propagation of the coating due to its high thermal stability (decomposition temperature at about 790°C) [7].

Sample D showed significant improvement in reducing fire propagation ((*I*) = 4.3) compared to samples A, B, and C. The appropriate combinations of Al(OH)_3_/CES/Mg(OH)_2_ with flame-retardant ingredients and acrylic binder in formulation D led to a highly effective fire stopping behavior.

The maximum temperatures of samples A, B, C, and D were 418°C, 391°C, 372°C, and 259°C, respectively. This result indicated that the sample D with appropriate combinations of flame-retardant ingredients and fillers caused the fire propagation hardly present compared to samples A, B, and C. This phenomenon is due to the synergist and catalysis effects of these three flame-retardant fillers (Mg(OH)_2_/Al(OH)_3_/CES) have chemical reaction with the fundamental ingredients (in general, the acid source, APP) on carbonaceous char formation [29–31], which improve the thermal stability and flame retardancy of the coating. Both Al(OH)_3_ (decomposition temperature at about 180°C) and Mg(OH)_2_ (decomposition temperature at about 330°C) hydroxides decompose endothermically when heated according to the reactions:

(4) $$\begin{array}{c} 2\text{Al}{\left(\text{OH}\right)}_{3}\left(\text{s}\right)⟶{\text{Al}}_{2}{\text{O}}_{3}\left(\text{s}\right)+3{\text{H}}_{2}\text{O}\left(\text{g}\right) \end{array}$$

(5) $$\begin{array}{c} \text{Mg}{\left(\text{OH}\right)}_{2}\left(\text{s}\right)⟶\text{MgO}\left(\text{s}\right)+{\text{H}}_{2}\text{O}\left(\text{g}\right) \end{array}$$

On exposure, Al(OH)_3_ has shown the strong reversibility of the dehydration reaction with water released inside the particle recombining with the reactive surface of the freshly formed alumina, resulting in a good flammability resistance filler [32]. For the endothermic decomposition of the Mg(OH)_2_ filler, the gaseous water phase is believed to envelop the flame, thereby excluding oxygen and diluting flammable gases [33].

However, coatings C and D exhibited a uniform expansion of the char layer (Figures 2(c) and 2(d)). The formation of a cohesive structure during burning can potentially be initiated by different phenomena such as the CO_2_ released due to the decarbonation of CES at about 790°C which could trap the degradation products into the residue and induce the swelling. It can be concluded that incorporation of CES acts as an additional blowing agent in reducing sample temperature for the coatings [7]. CES containing 95% CaCO_3_ releases noncombustible gases (carbon dioxide) on heating, to form calcium oxide as follows [34]:

(6) $$\begin{array}{c} \text{Ca}{\text{CO}}_{3}\left(\text{s}\right)⟶\text{CaO}\left(\text{s}\right)+\text{C}{\text{O}}_{2}\left(\text{g}\right) \end{array}$$

### 3.2. Surface Micrographs of the Foam Structures

**Figure 3:**
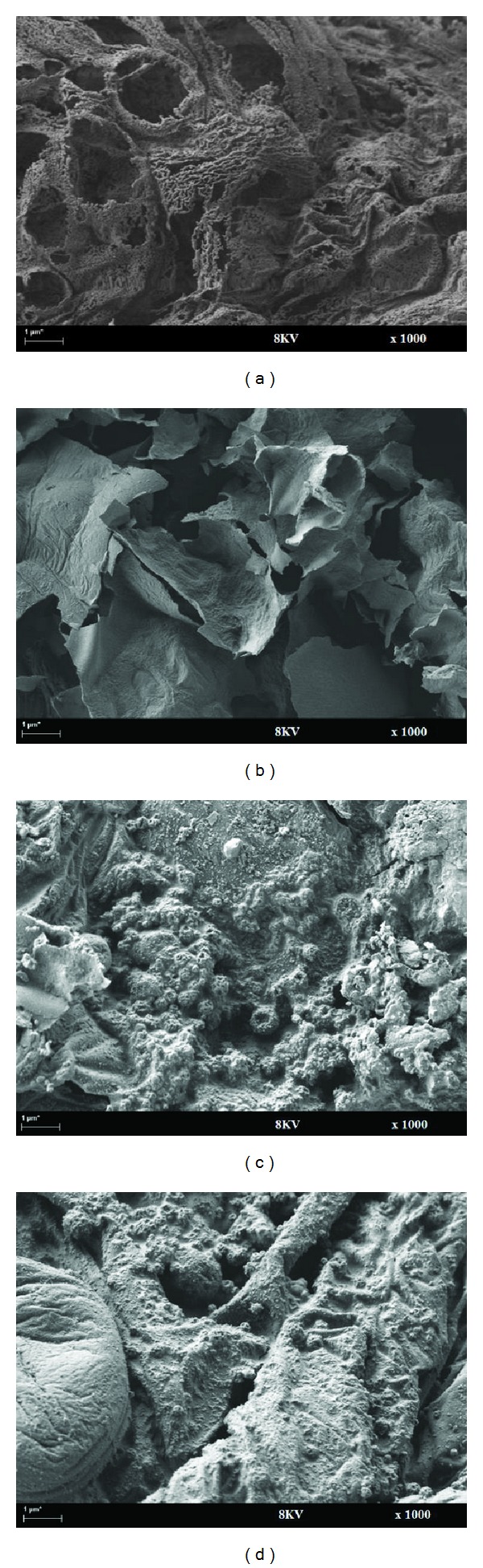
Surface micrographs of the foam structure of samples A, B, C, and D.

High magnification surface micrographs enable the observation of the surface morphologies of form structure formation as shown in Figure 3.

It was found that the foam structure of samples C and D was significantly improved by filling CES biofiller which produced denser and more uniform foam structure compared with samples A and B, which are porosity, nonuniform, and tiny form structure. The fire protection performance indicated that the efficiency of the char layer to fire-resistance depended strongly on its physical structure [27]. The char layer of D had more uniform and dense foam structure, which could isolate steel substrate from fire and provide better fire protection. Moreover, the char layers of A and B had porous form structure with high porosity observed. The tiny foam structure could insulate steel substrate from heat and fire. However, heat might transfer to the steel substrate through the porous foam structure, which could lead to a decline of fire protection. The char layer of B showed a broad distribution of the cell size. This foam structure demonstrated that some cells burst, which could increase efficiency of heat transfer and decline of fire protection.

### 3.3. Thermal Analysis of the Coating Samples

**Figure 4:**
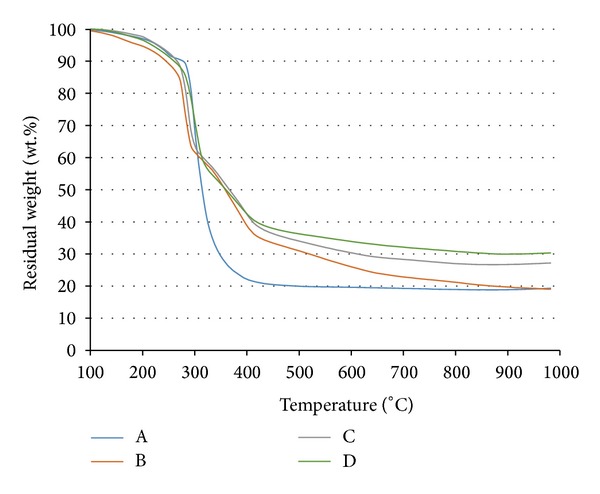
TGA curves of samples A, B, C, and D.

Thermal degradation of samples A, B, C, and D was analyzed using the TGA test (Figure 4). The curves of the coatings were similar between 100°C and 285°C and weight loss of each coating was less than 34 wt.% at 285°C, whereas the thermal degradation of the samples left a thermally stable char at 900°C.

When the temperature was higher than 300°C, the TGA curves of the coatings became slightly different from each other. The total weight losses of samples A, B, C, and D were about 81.6%, 80.8%, 73.9%, and 68.7%, respectively. The residual weights difference of fillers/flame-retardant additives/binder composites, which could reveal the possible reaction and reaction temperature between fillers and flame-retardant additives or the binder resin. The TGA curve of C showed that the residue weight of the coating was increased by adding CES filler compared with coatings A and B due to its higher decomposition temperature of CES contains (790°C) [7]. The highest residue weight of coating D indicated that the combination of Mg(OH)_2_/CES/Al(OH)_3_ could significantly enhance thermal stability and antioxidation of the coating. It was found that the presence of Mg(OH)_2_/CES/Al(OH)_3_ fillers which led to thermal stabilization of coating is remarkably improved in the temperature range of 350–1000°C. It means the reaction between fillers with flame-retardant additives showing a synergism behavior [35].

### 3.4. Static Immersion Test

**Figure 5:**
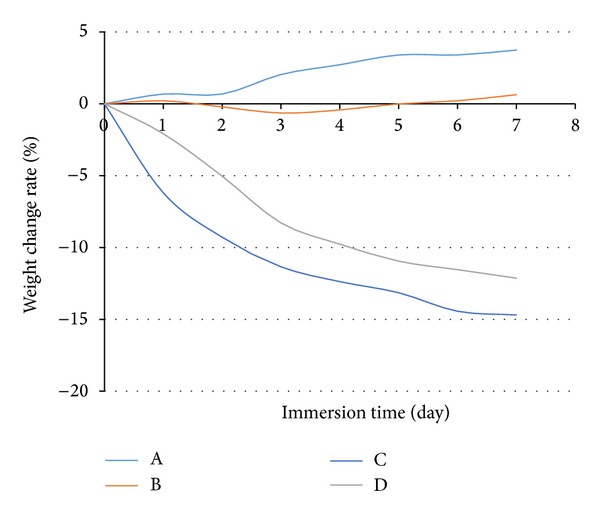
Relationship between weight change rate and immersion time for different coating in water.

The weight change rate curves of the thin-film coatings are shown in Figure 5. It was observed that the water could destroy some components of hydrophilic flame-retardant ingredients and break some bonds of binder, so the water resistance of intumescent coatings decreased significantly.

When samples A, B, C, and D were immersed in water for 7 days, two main processes (permeation and migration) took place simultaneously for sample B. However, the migration process occurred for samples C and D, which contain CES filler. Moreover, some hydrophilic flame-retardant ingredients might migrate from coating and be solved in water during the migration process, which resulted in weight loss of coating  [36]. In the permeation process, water could infiltrate into the pore structure of the coating, which led to the increase of weight of the sample.

The experimental results show that the weight of coatings A was gradually increased and its weight gain rates were about 4.08% at 7 days due to the fact that the permeation process of water exceeded the migration process of fire retardant ingredients. However, the weight of coatings C and D was gradually decreased and reached equilibrium at 6 days due to the fact that the migration process occurred, and their weight loss rates were −12.13% and −14.69%, respectively, at 7 days.

It can be concluded that incorporation of Al(OH)_3_ to flame-retardant additives and acrylic binder could slow down permeation of water and migration of fire retardant ingredients due to its poorly solubility in water [37], which led to an improvement in resisting water permeation and migration ability of the coating. Weight loss rate and weight gain, weight of all samples, were maintained relatively constantly at 6 days of the test. The cracking and blistering phenomena did not occur in all samples after 7 days of the test.

### 3.5. Adhesion Strength

**Figure 6:**
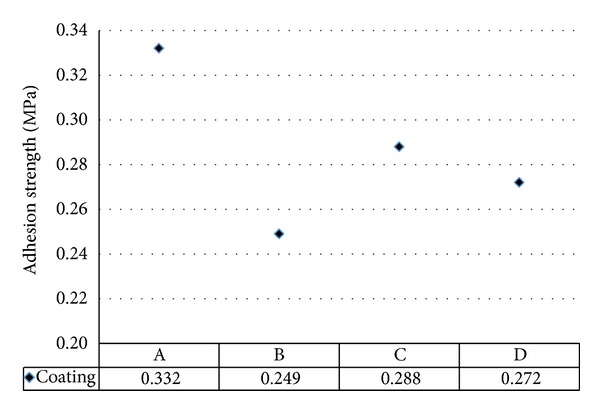
The adhesion strength of coatings A, B, C, and D.

Table 3 displays the average adhesion strength values of the coating samples. The adhesion strength of coatings A, B, C, and D was 0.332 MPa, 0.249 MPa, 0.288 MPa, and 0.272 MPa, respectively (Figure 6).

Coating B had poor adhesion strength of 0.249 MPa compared with coatings A, C, and D, which had values of 0.332 MPa, 0.288, and 0.272 MPa, respectively. An increase in the adhesion strength of the coating A filled by Mg(OH)_2_ filler is due to the strong bonding strength between the metal surface and acrylic binder/Mg(OH)_2_ filler interface adhesion for effective stress transfer [38]. However, incorporation of CES filler in coating C increased the adhesion strength by 15.7% compared to coating B. The improvement in the adhesion strength is due to the better reinforcement properties between CES filler and acrylic binder [39]. This result indicated that the incorporation of Al(OH)_3_ filler in coatings B and D had decreased the adhesion strength compared with coatings A and C without addition of Al(OH)_3_ filler. In conclusion, choosing appropriate flame-retardant filler could strongly influence the bonding strength of intumescent coating.

## 4. Conclusions

This study has concluded that the intumescent flame protective coatings have been found to be quite effective on fire resistance performance. On exposure, the coating provided multicellular insulating foam that acted as an effective barrier in the conduction of heat into the steel substrate. The appropriate combinations of Al(OH)_3_/Mg(OH)_2_/CES flame-retardant fillers in flame-retardant ingredients and binder reduced the fire propagation index, while possessing good water repellency, char formation, thermal stability, and adhesion. Addition of CES as a biofiller showed significant improvement in fire protection, which has a great potential for use as ecofriendly filler while at the same time it preserves the environment. Hence, this study reveals that the appropriate combinations of flame-retardant ingredients results proved that using this intumescent coating on steel structures is a feasible fireproof material to maintain the structure properties in a fire.

## Figures and Tables

**Table 1:** Compositions of intumescent fire protective coatings.

Ingredients	Parts by weight for formulations			
	A	B	C	D
APP	18.00	18.00	18.00	18.00
MEL	9.00	9.00	9.00	9.00
PER	9.00	9.00	9.00	9.00
TiO_2_	5.50	5.50	5.50	2.75
Al(OH)_3_	—	5.50	—	2.75
Mg(OH)_2_	5.50	—	—	2.75
CES	—	—	5.50	2.75

Binder				
Acrylic resin	53.00	53.00	53.00	53.00

**Table 2:** Results of fire propagation test.

Time (min.)	Calibration	Specimen A	Specimen B	Specimen C	Specimen D
		AR + T + M	AR + T + A	AR + T + CES	AR + T + M + A + CES
0.5	14	18	12	14	12
1	18	21	15	18	16
1.5	23	26	19	23	19
2	27	30	22	27	23
2.5	30	34	25	31	26
3	34	38	30	34	30
4	72	122	87	55	54
5	108	212	149	133	129
6	129	274	179	169	181
7	148	321	223	243	202
8	166	364	312	327	219
9	182	378	336	372	234
10	192	405	372	360	244
12	214	417	391	355	249
14	230	418	370	349	253
16	238	416	326	310	258
18	246	403	298	299	260
20	257	384	281	290	263

Subindex 1		1.8	0	0.2	0
Subindex 2		15	8.4	8.3	3.8
Subindex 3		5.7	3.4	2.9	0.7
Index of performance		**22.3**	**11.5**	**11.2**	**4.3**

**Table 3:** Adhesion strength of coatings.

Coating	Crack charge, *F* (N)	Sticking area, *A* (m^2^)	Adhesion strength, *f*_*b*_ (MPa)
A	830	0.25 × 10^−2^	0.332
B	623	0.25 × 10^−2^	0.249
C	720	0.25 × 10^−2^	0.288
D	680	0.25 × 10^−2^	0.272
